# Transient cartilages and interdigital vasculature organization in the embryonic duck foot

**DOI:** 10.1098/rsos.260583

**Published:** 2026-09-09

**Authors:** João F. Botelho, Ignacio Casanova Maldonado, Macarena Faunes, Verónica Palma, Bhart-Anjan S. Bhullar

**Affiliations:** ^1^ Escuela de Medicina Veterinaria, Facultad de Agronomía y Sistemas Naturales, Facultad de Ciencias Biológicas, Facultad de Medicina, Pontificia Universidad Católica de Chile, Santiago, RM, Chile; ^2^ Millennium Nucleus Early Evolutionary Transitions of Mammals, ANID, Chile; ^3^ Departamento de Biología, Facultad de Ciencias, Universidad de Chile, Santiago, Chile; ^4^ Department of Geology and Geophysics, Yale University, New Haven, CT, USA

**Keywords:** avian evolution, vasculature, webbed foot, reactive oxygen species, programmed cell death, Turing reaction

## Abstract

The evolution of webbed limbs represents a recurrent adaptation to aquatic life in tetrapods, facilitated by the dynamics of embryonic digit development. Digits form at the distal tip of the limb bud as cartilage condensations surrounded by mesenchymal cells, a process described as a Turing-type reaction between activator and inhibitor molecules. In species with free digits (lacking interdigital membranes), extensive programmed cell death occurs in the interdigital tissue, whereas this process is reduced in species with webbed limbs. Other factors potentially involved in the development of webbed limbs are fluctuations in oxygen tension, production of reactive oxygen species, and vascular remodelling, all of which have been documented in the embryonic interdigital tissue. Whether and how these phenomena are interrelated to the regression or persistence of the interdigital membrane remains poorly understood. On the basis of the analysis of skeletal and vascular development in ducks, using whole-mount immunofluorescence, we identified transient populations of chondrocytes that appear to influence the maintenance of interdigital vasculature and permit the continued growth of the membrane. Finally, we propose that current Turing models can explain the formation of these transient chondrocytes in ducks.

## 1. Introduction

In every major lineage of terrestrial vertebrates, some species have evolved into secondarily aquatic forms [1]. Among the adaptations for swimming, the most common is the development of interdigital webs connecting the digits into a fin-like structure that increases propulsion underwater [2]. Webbed limbs have evolved independently in mammals, birds, lizards, turtles, and crocodilians multiple times. In birds alone, webbed feet have evolved at least eight times [3,4,5].

Conserved developmental mechanisms underlying digit formation enable the recurrent evolution of webbed limbs [6,7]. Digits arise from mesenchymal condensations in the distal limb bud. Each condensation generates a cartilage—first the metapodials, then the phalanges in a proximal-to-distal sequence. When the final phalanges develop in the distal tip of each digit, the digits are still connected by interdigital tissue. Extensive programmed cell death (PCD) occurs in the interdigital tissue of non-webbed species such as chickens [8], mice [9], and lizards [10]. By contrast, in webbed species secondarily adapted to aquatic life, such as turtles, ducks, and coots, interdigital PCD is reduced, supporting the view that PCD sculpts the interdigital tissue during development [11,12]. Further studies have shown that inhibition of PCD by interfering with fibroblast growth factor (FGF) [13], bone morphogenetic protein (BMP) [14–16], and retinoic acid [17,18] pathways results in syndactyl embryos.

The interdigital tissue comprises undifferentiated mesenchymal cells covered by a thin epidermis and irrigated by a dense network of vessels. This vascular network undergoes two massive remodelling processes, first when digit mesenchymal condensations generate avascular cartilages [19,20], and then when vessels disappear in the interdigits of chicken and mouse embryos, but not in ducks [21]. Vascularization is essential for tissue growth in normal and pathological conditions, and several lines of evidence suggest that reduced growth rates of interdigital tissue in relation to digit elongation are the main cause for the development of free digits in amniotes [22,23]. Recently, reactive oxygen species (ROS) have been linked to PCD of the interdigital tissue, calling attention to the possible causal role of oxygen levels and the vasculature [24–27]. Lower atmospheric oxygen levels or antioxidant molecules reduce ROS-mediated PCD in mice and chickens, whereas experimental increase of blood vessel density promotes it [24–26].

Digit development is influenced by two organizing centres: the zone of polarizing activity (ZPA) [28], composed of posterior mesenchymal cells that secrete Sonic hedgehog (Shh) [29], and the apical ectodermal ridge (AER) [30], formed by epidermal cells that secrete different FGF [31–33]. AER and ZPA activities drive limb distal elongation and anteroposterior widening, respectively. Together, they set the limb field size in which an alternating series of digits and interdigits develop by a self-organized Turing-like mechanism [34,35]. Some molecules proposed for this mechanism are also involved in the control of PCD during limb development. For instance, an activator/inhibitor system generates alternating stripes of cells expressing either SOX9 or wingless-related integration site (WNT) and BMP pathway molecules [36,37], and several experiments have demonstrated that the BMP pathway is necessary for PCD in the interdigit [38–44].

To further explore the relationship between PCD, cell proliferation, vascular remodelling, and skeletal development in the webbed foot of ducks, we used whole-mount immunofluorescence to visualize the embryonic limb vasculature and skeletal elements, as well as the distribution of proliferative, apoptotic, and senescent cells. On the basis of these data, we propose a hypothesis integrating digit patterning with the evolution and development of the interdigital membranes.

## 2. Material and methods

Domestic chicken (*Gallus gallus*) and domestic duck (*Anas platyrhynchos*) fertile eggs were purchased in local farms and incubated at 37.5°C and 60% humidity until the desired stage. All experimental procedures were conducted in accordance with Chilean regulations and approved by the Institutional Animal Care and Use Committees (CICUA) of the Pontifical Catholic University of Chile.

### 2.1. Neutral red and dihydroethidium staining

Right limbs were placed in a 5 cm diameter Petri dish containing neutral red (NR) vital staining (Thermo Fisher Scientific, N3246, 4 mg ml^−1^) diluted in pre-warmed 1X Dulbecco's modified eagle medium (DMEM) at 37°C for 30 min in darkness to evaluate the distribution of cell death. The autopods were washed in 1x phosphate buffered saline (PBS) and immediately photographed using a dark-field stereomicroscope. Left limbs were placed in pre-warmed DMEM containing dihydroethidium (DHE; Sigma-Aldrich D7008, 1 µM) at 37°C for 30 min to detect ROS. The autopods were washed in 1x PBS and photographed using a fluorescence stereomicroscope. Images were digitally inverted for comparison with right limbs.

### 2.2. Whole-mount immunofluorescence

Embryonic legs were dissected in cold 0.1 M PBS and fixed in 4% paraformaldehyde (PFA) at 4°C for 24 h. Samples were washed twice in PBS (15 min each), dehydrated in methanol, and stored at –80°C. Embryos were incubated overnight at room temperature in 80% methanol/10% H₂O₂/10% dimethyl sulfoxide (DMSO), washed in 100% methanol, and rehydrated through 50% methanol/PBS to PBS. They were then transferred to CUBIC solution (25% urea, 10% tetrakis, 5% Triton X-100) and cleared at 37°C for 2–3 days under gentle agitation. After washing in PBS with Triton X-100 (PBS-T; 0.5% Triton X-100), samples were blocked for 2 h in PBS-T supplemented with 10% horse serum and 5% DMSO. Primary antibodies were applied in PBS-T containing 5% serum, 5% DMSO, and 0.1% sodium azide for 1–4 days at room temperature with agitation. After washing in PBS-T (2 × 15 min, then 4 × 1 h), samples were incubated with secondary antibodies in PBS-T containing 5% DMSO and 0.1% sodium azide for 1–2 days. Following immunolabelling, samples were washed again (2 × 15 min, 4 × 1 h) and transferred to CUBIC mounting solution (50% sucrose, 25% urea, 25% tetrakis). The following antibodies were used: collagen type 2a (Developmental Studies Hybridoma Bank, 1 : 20), Sox9 (Merck Millipore, 1 : 500), phosphorylate SMAD 1/5 (Cell Signalling Technology, 1 : 500), phosphorylated histone-3 (Cell Signalling Technology, 1 : 500), cleaved caspase-3 (Cell Signalling Technology, 1 : 500), wheat germ agglutinin lectin (VectorLab, 1 : 1000), anti–mouse Alexa-488 (1 : 300, Invitrogen), and anti-rabbit Alexa-568 (1 : 300, Invitrogen).

### 2.3. Embryonic vasculature labelling and rendering

Fertile chicken and duck eggs were incubated horizontally for 3 days, after which 3.5 ml of albumen was removed from the blunt end to lower the embryo. At the desired stages, a shell window was opened to expose the vasculature. Microcapillary needles (15–20 µm tip) were loaded with 10 µl fluorescein-labelled wheat germ agglutinin (1 µg ml^−1^; Vector S.A.) and injected into the chorioallantoic membrane using a mouth pipette. Eggs were incubated for 15 min to allow complete vascular labelling, after which embryonic legs were dissected in 0.1 M PBS and fixed in 4% PFA at 4°C for 24 h. Samples were processed for whole-mount labelling and imaging as described above. For three-dimensional rendering of immunofluorescence, confocal stacks were transformed into image sequences and loaded into Acto3D software [45].

## 3. Results

### 3.1. Reactive oxygen species production is not related to vascular regression in the embryonic foot of ducks

**Figure 1.**
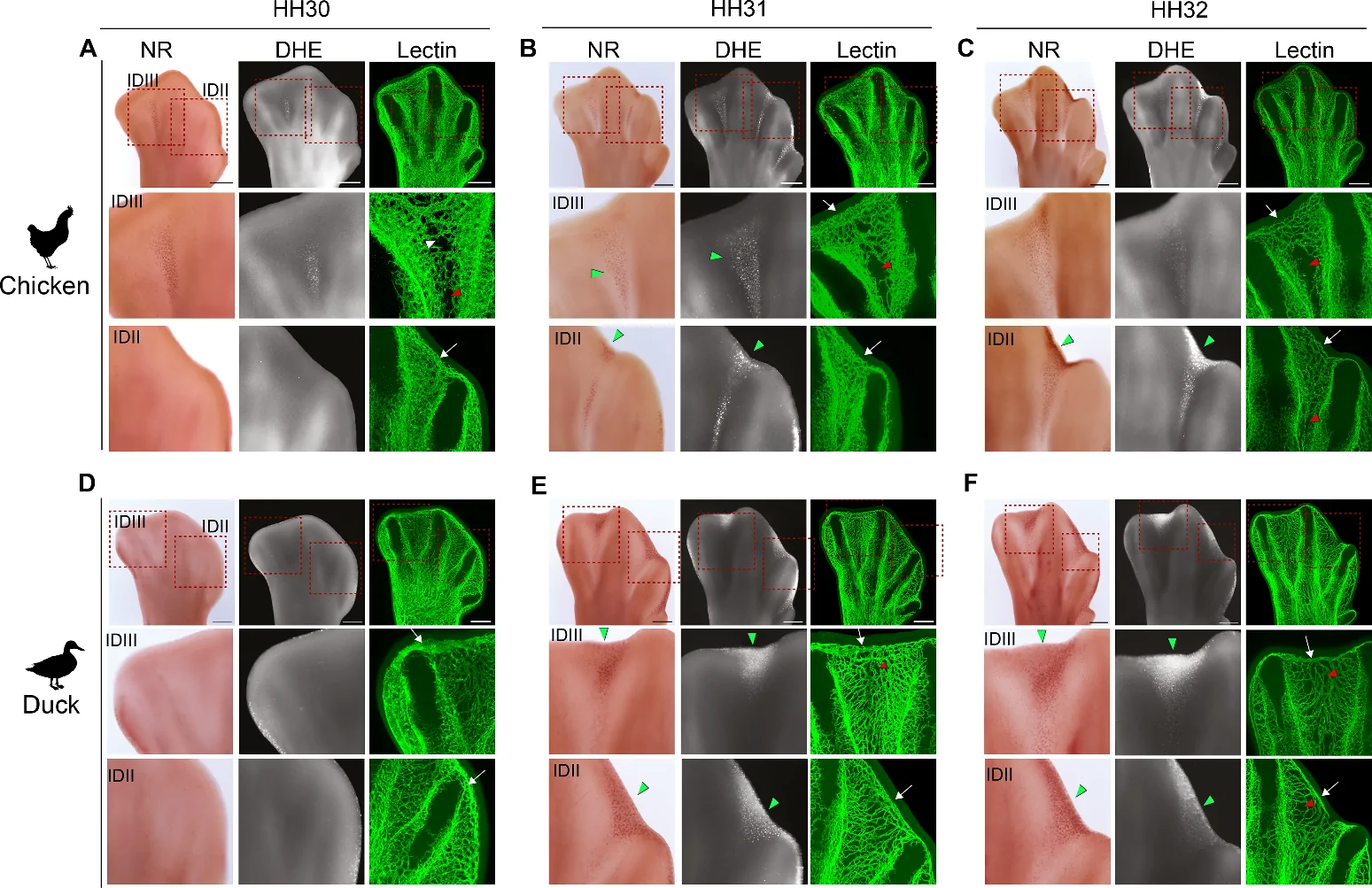
PCD, ROS, and vasculature remodelling in chick and duck embryonic feet. Whole-mount detection of PCD, ROS, and vasculature in chicken (A–C) and duck (D–F) feet at stages HH30–HH32. Columns show NR (PCD), DHE (ROS), and lectin staining (vasculature). The first row in each panel shows the entire foot at low magnification, and the second and third rows display higher-magnification views of interdigits III and II, respectively. Green arrowheads mark regions of overlapping PCD and ROS, red arrowheads indicate vascular reduction, white arrowheads indicate constricted vessels, and white arrows indicate the vascular marginal sinus; scale bars, 500 µm.

**Figure 2.**
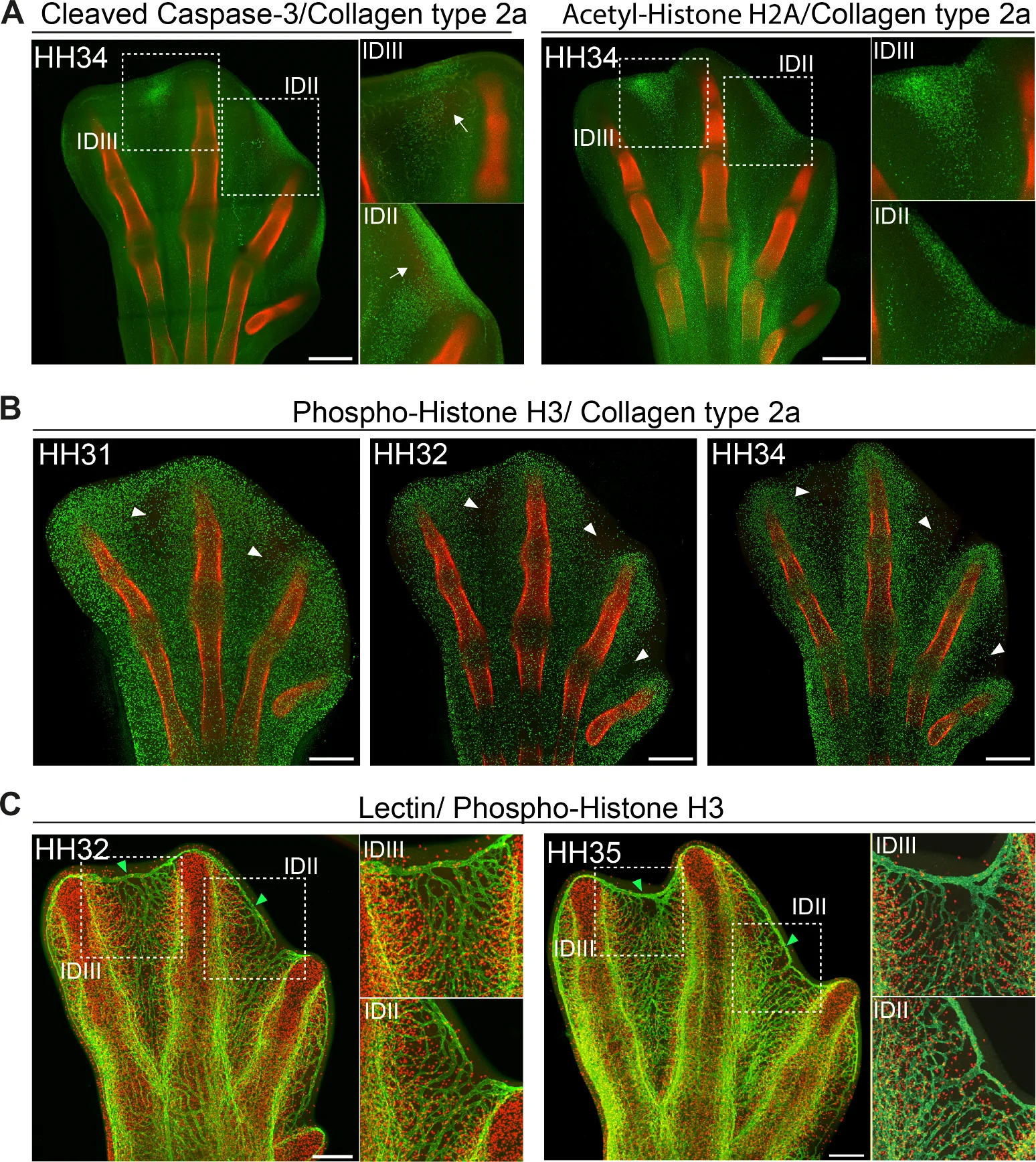
Apoptosis, senescence, and proliferation dynamics in the developing duck interdigital tissue. Whole-mount immunofluorescence for cleaved caspase-3 (apoptosis marker), acetyl-histone H2A (senescence marker), phospho-histone H3 (PH3; proliferation marker), collagen type 2a, and lectin (vasculature) in duck embryonic feet at stages HH31–HH35. (A) Cleaved caspase-3 (green, left), acetyl-histone H2A (green, right), and collagen type 2a (red) at HH34. Insets show higher-magnification views of interdigits II and III. (B) PH3 (green) and collagen type 2a (red) staining from HH31 to HH34. (C) Lectin (green) and PH3 (red) staining at HH32 and HH35. White arrowheads indicate collagen type 2a-positive cell populations within interdigits; white arrows mark regions of reduced cell proliferation; green arrowheads denote the vascular marginal sinus; scale bars, 500 µm.

**Figure 3.**
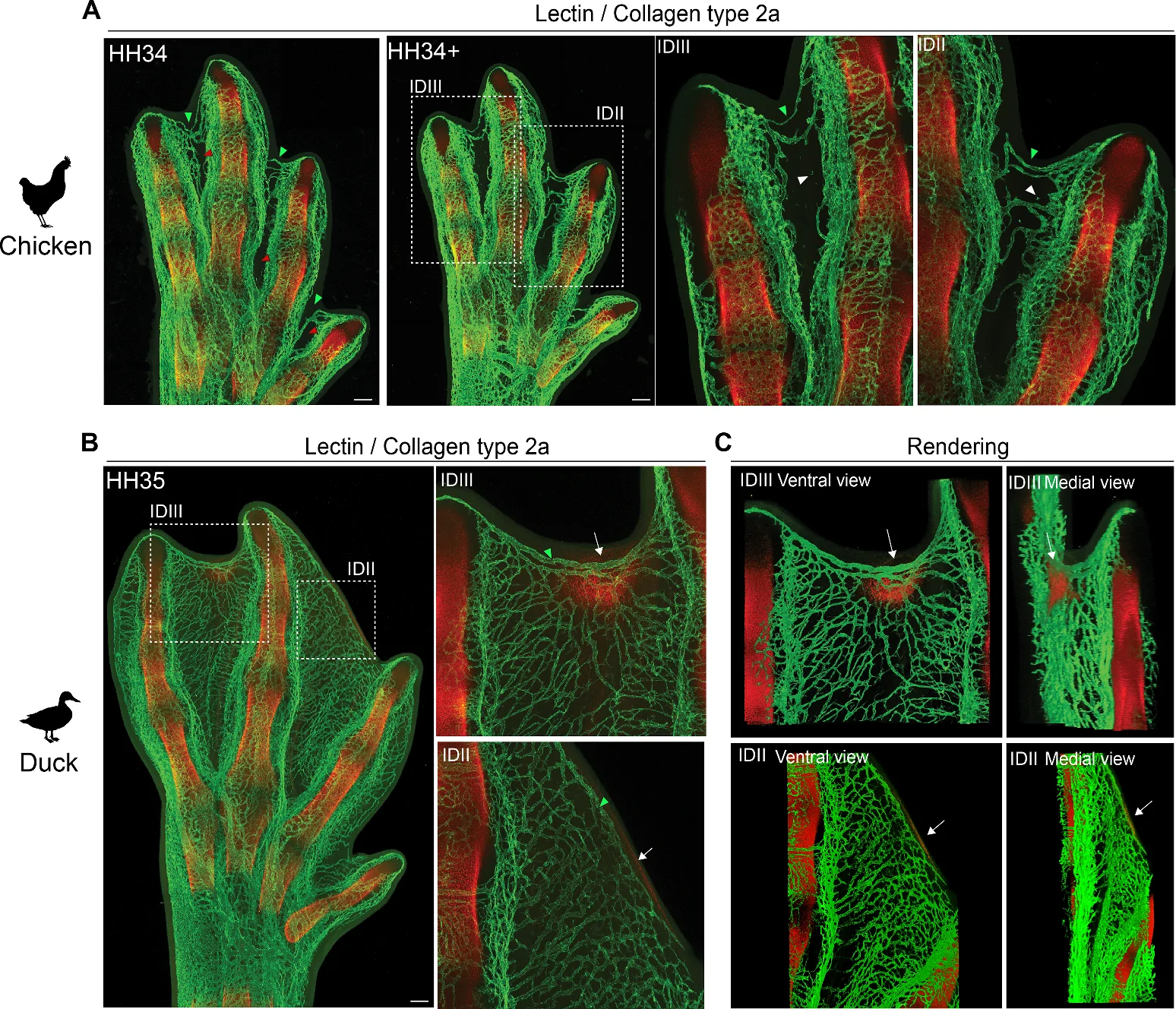
Organization of the vasculature in chicken and duck embryonic feet. Whole-mount immunofluorescence for lectin (vasculature, green) and collagen type 2a (red) in chicken and duck embryos at stages HH34–HH35. (A) Chicken feet at HH34 and HH34^+^, and higher-magnification views of interdigits III and II. (B) Duck feet at HH35. Insets show higher-magnification views of interdigits III and II. (C) Three-dimensional renderings of interdigits III and II from (B). Ventral (right) and medial (left) views. Green arrowheads in (A) and (B) point to the vascular marginal sinus; white arrowheads in (A) highlight vasculature regression in the chicken interdigits, and white arrows in (B) and (C) indicate collagen type 2a signal within interdigits; scale bars, 500 µm.

PCD does not normally occur in the interdigit of *Xenopus* tadpoles [10,46], but it does in tadpoles raised under atmospheric oxygen levels, raising the possibility that in the evolutionary transition from aquatic to terrestrial environments, increased oxygen concentrations may have contributed to the development of free digits through the production of ROS. To investigate how a webbed-foot amniote deals with ROS under atmospheric oxygen levels, we analysed duck embryos using NR and DHE to detect PCD and ROS, and vitelline vein lectin injections followed by whole-mount confocal imaging to reconstruct vasculature [47]. We compared the results with non-webbed chicken embryos and found that in both species, PCD and ROS production are correlated (figure 1, green arrowheads) [48]. In both species, ROS production occurs in regions containing a dense vascular plexus and is followed by a reduction in vessel density (figure 1, red arrowheads). In chickens, vascular remodelling leads to the formation of avascular zones in all interdigits, first in the interdigit III (figure 1A–C), and later in the interdigits I and II (figure 3A, red arrowheads), whereas ducks retain a less dense, yet persistent, vasculature in the interdigits II and III (figure 1D–F, red arrowheads). ROS activity in the interdigit has also been associated with caspase-mediated apoptosis and cellular senescence [49]. Whole-mount immunofluorescence for cleaved caspase-3 and acetylated histone H2A in duck embryos revealed that apoptosis and senescence take place in regions of high ROS production (figure 2A,B). These findings suggest that, unlike aquatic amphibians, ducks do not suppress ROS activity during webbed-foot development but rather harness it to remodel and maintain a functional interdigital vasculature.

### 3.2. The vascularized interdigital tissue of ducks shows a marked decline in cell proliferation

**Figure 4.**
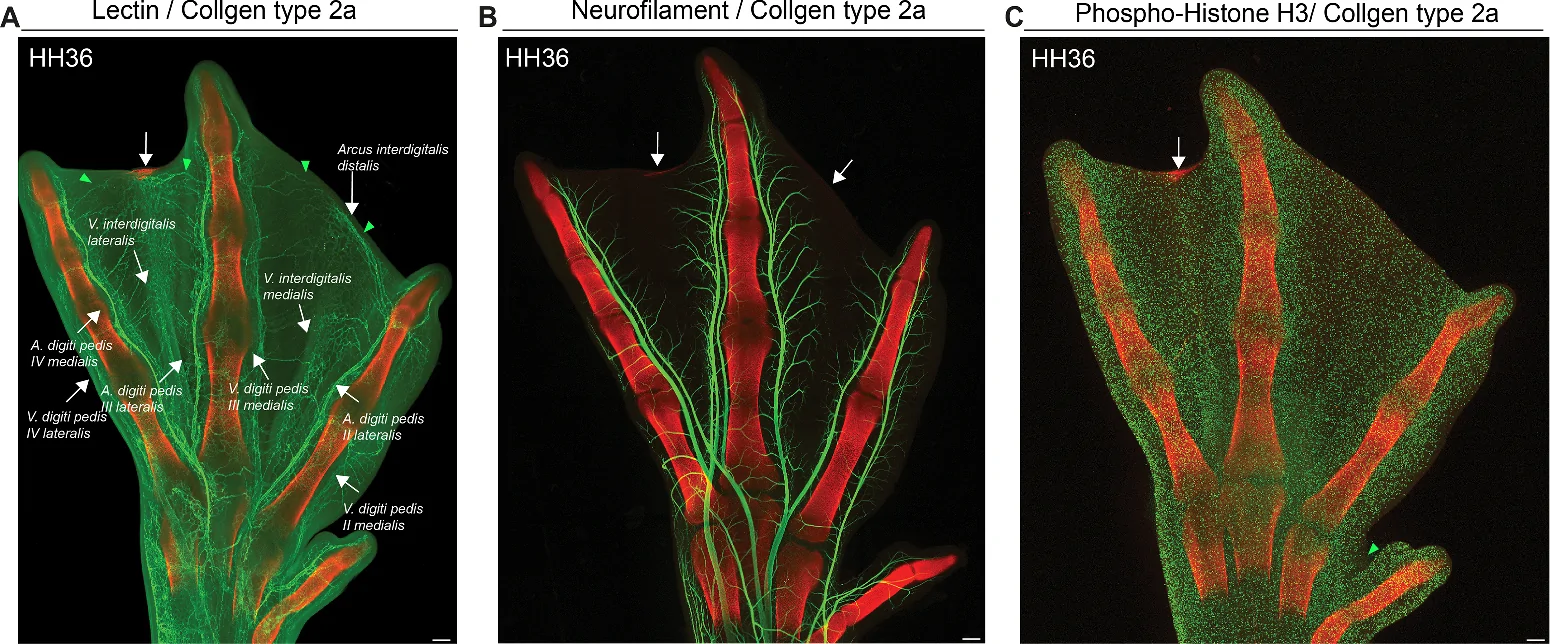
Interdigital veins, digital nerves, and cell proliferation in the duck foot at HH36. (A) Immunofluorescence against collagen type 2a (red) and lectin labelling of vasculature (green). (B) Immunofluorescence against collagen type 2a (red) and neurofilament (green). (C) Immunofluorescence against collagen type 2a (red) and phospho-histone H3 (green). White arrows in (A) point to developing digital arteries and interdigital venae. Note that interdigital veins develop in non-innervated areas with high levels of cell proliferation; scale bar, 500 µm.

In chicken and mouse embryos, vascular regression in the interdigital region is accompanied by a substantial decrease in the number of proliferating cells (supplementary material, figure S1) [50,51], resulting in undergrowth of interdigital tissue in relation to digits [22,23]. Whole-mount PH3 immunofluorescence in duck limbs revealed that, despite the presence of a complete vascular network, proliferation also decreases in all interdigital regions from stages HH32 to HH34 (figure 2B,C) and then recovers at HH35 in all but the non-webbed interdigit I (figures 2C and 4C). The areas of reduced proliferation expand from distal to proximal and are not initially correlated to PCD and ROS production areas (figure 2B,C, white arrows), which inversely expand from proximal to distal in ducks (figure 1) [48].

During early development, a marginal vein surrounds the dorsoventral border of the limb bud [52]. In the autopod, it eventually forms a vascular sinus that underlies the highly proliferative avascular mesenchyme under the AER [53,54]. In chicken, the vascular sinus disappears around HH35 (figure 3A, green arrowheads). In ducks, a thick vascular sinus, in an area of strong ROS production, gives rise to vascular arches along the margin of the interdigits II and III, from which later interdigital veins extend proximally [55,56]. The interdigit I does not form a vascular arch (figure 3B). The development or regression of interdigital vascular arches does not temporally coincide with the presence or absence of the overlying AER [57].

### 3.3. Ducks have transitory cartilages in the interdigit

Previous ultrastructural examination of the interdigit III of the developing duck foot showed deposition of collagenous material at the distal epithelial/mesenchymal interface [11]. Using whole-mount immunofluorescence, we identified collagen type IIα–positive cells resembling chondrocytes within the distal regions of interdigits II and III. These cells first appear as a sparse population located anterior to the marginal vascular arches (figure 2A, white arrows) and later produce small patches of collagen fibres aligned parallel to the membrane surface (figures 3B and 4A, white arrows). Notably, in poorly perfused lectin injections, arteries, but not veins, are distinctly labelled. In such cases, distal branches of the *arteriae digiti pedis* exhibit a clear orientation towards the collagen patches in interdigits II and III (figure 4A), where the returning flow occurs through nascent *venae interdigitale*.

Developing axons can serve as templates for arterial differentiation. To rule out the possibility that the development of interdigital vasculature follows its innervation, we immunolabelled developing axons and terminals. As in chicken embryos (supplementary material, figure S1B), duck foot innervation is initially restricted to the digit periphery, and it later extends into the interdigital membranes through the lateral and medial branches of digital nerves (figure 4B). Thus, interdigital vasculature does not follow developing nerves.

## 4. Discussion

The chondrogenic potential of the interdigital tissue is well supported by numerous experimental manipulations that induce the formation of ectopic digits within it. These include ectopic expression of *Noggin* in the AER [58], implantation of beads soaked in transforming growth factor beta [39], treatment with Janus Green B [59], high doses of bromodeoxyuridine [60], foil barriers in the interdigit [61], and partial removal of the AER (supplementary material, figure 1C) [62,63]. Turing-like mechanisms currently invoked to explain digit patterning based on alternating stripes of digit and interdigit signalling molecules also provide a useful framework for understanding the experimental formation of interdigital cartilages. As the autopod grows, these stripes spread apart distally, increasing the distance between the tips of adjacent rays [64]. To prevent stripe bifurcation or segmentation, the wavelength must increase distally, a process modulated by distal *Hox* gene dosage and dependent on limb pad size [64,65]. Notably, some *in silico* simulations create ectopic patches of SOX9 expression in the distal interdigit [37]. Therefore, experimental manipulations or natural variation in limb pad width or distal HOX gene dosage could drive the Turing-like network to generate small patches of interdigital cartilage.

Cartilage development begins with the formation of an avascular mesenchymal condensation. Once chondrocytes differentiate, they secrete angiogenic factors that promote the formation of a dense vascular network surrounding the cartilage [19]. Vessel perfusion is also essential for vascular remodelling, with regression occurring in non-functional vessel segments and angiogenesis taking place in regions of high blood flow. Importantly, vascular regression is not driven by endothelial cell death but rather by coordinated endothelial proliferation, reorientation, and migration in response to hemodynamic forces (see lumens constricted in figure 1A, white arrowhead) [66].

**Figure 5.**
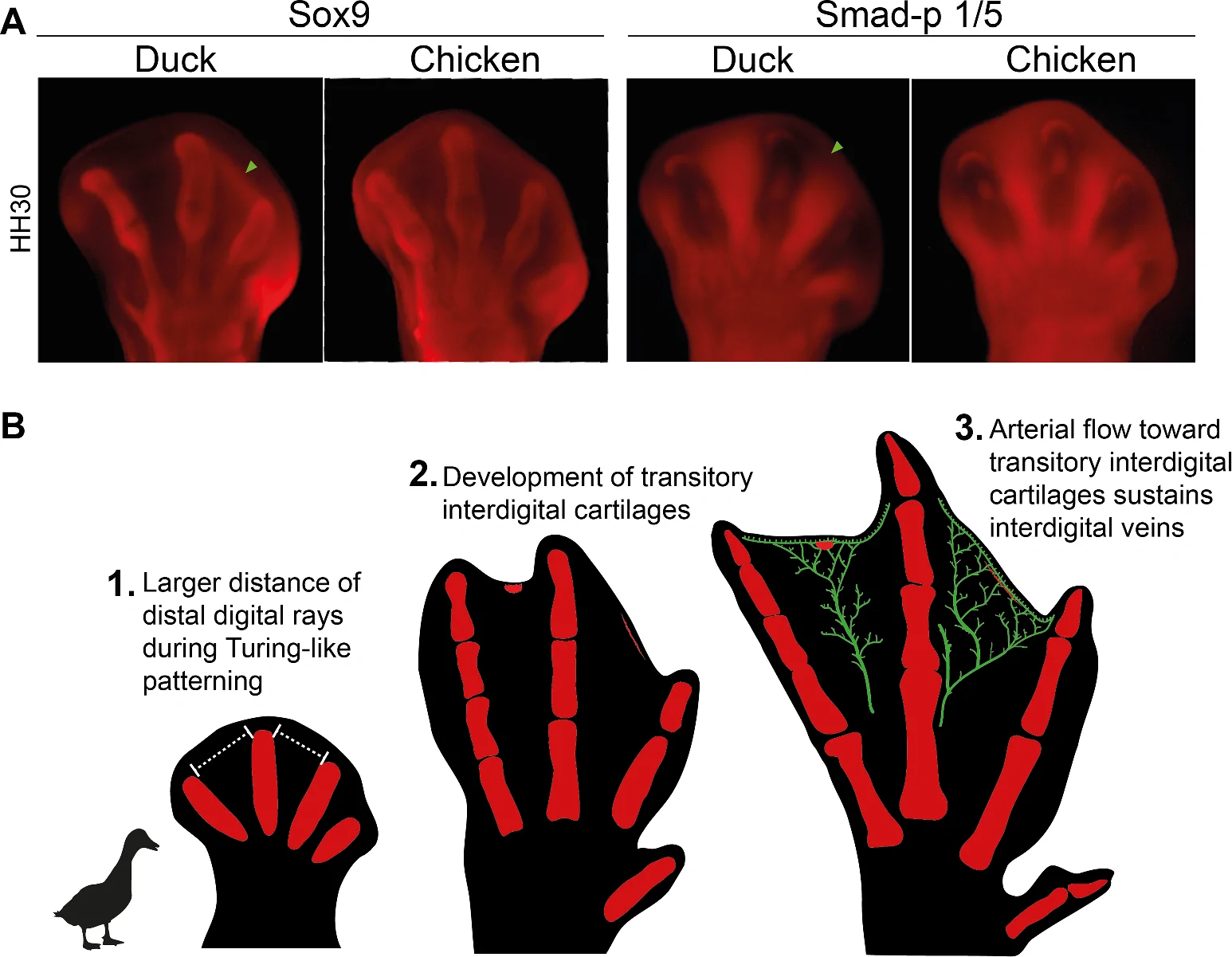
A model for webbed-foot development in ducks: (A) whole-mount immunofluorescence in duck and chick feet at HH30 showing interdigital production of Sox9 and the absence of Smad-p 1/5 in the interdigit II of ducks (green arrowheads); and (B) a mechanistic model for the development of webbed avian feet. Large distances between digital rays at distal positions promote the production of transitory interdigital cartilages via Turing-like mechanisms. These cartilages induce arterial irrigation, which in turn sustains the presence of interdigital veins; scale bars, 500 µm.

The arrest of cell proliferation during foot development in ducks shown here suggests that the reactivation of proliferative activity could play a central role in webbed-foot formation. We hypothesize that the arrest and subsequent recovery of cell proliferation might result from changes in vessel perfusion. Initially, most vessels concentrate around the avascular digital cartilages, driving the remodelling of interdigital vasculature. Transient interdigital chondrocytes, generated by the radial expansion of SOX9 expression stripes in the broader duck limb pad (figure 5A), would attract blood vessels towards the distal margin of interdigits II and III, establishing a new interdigital blood flow that restores proliferative activity (figure 5B). In non-webbed species such as the chicken, interdigital vessels are instead drawn towards the digital cartilage, leaving the interdigit poorly perfused. Notably, the proximal region of interdigit II in chickens retains a small, vascularized membrane that regains proliferative activity around stage HH36 (supplementary material, figure 1B). Our hypothesis also helps explain why all palmated birds have a short hallux, whereas totipalmated birds, a morphology that evolved independently in tropicbirds, pelicans, and gannets/cormorants, possess long halluces [67]. A larger digit I anlage during early limb patterning, around stage HH30, would increase the distance between the tips of digits I and II, thereby promoting the formation of transient cartilages and interdigital vasculature.

Decades of research on autopod growth, patterning, and apoptosis have provided multiple examples of how developmental mechanisms can drive evolutionary adaptations [57,67–70]. The convergent evolution of webbed feet represents a paradigmatic example of it. The prevailing view holds that PCD of mesenchymal cells—modulated by ROS, oxygen tension, and BMP signalling—leads to the regression of interdigital tissue in non-webbed–footed species. Accordingly, webbed-footed species must evade this process to maintain interdigital growth in coordination with digital development. In this study, we suggest that the interaction between the skeletal and vascular system might allow duck embryos to keep interdigital growth, that ROS-mediated PCD regulates vascular density, and that the BMP pathway could influence interdigital PCD through its role in shaping mesenchymal condensations. Further studies should address whether transient cartilages are sources of angiogenic factors such as vascular endothelial growth factor, whether the development of ectopic SOX9-positive cells is directly influenced by the hypoxic environment [71,72], and whether there are differences in the behaviour of the Turing-like network in ducks and other web-footed species compared with non-web–footed birds.

## Supplementary Material



## Data Availability

Supplementary material is available online [73].
